# Role of Cardiac Rehabilitation in Heart Failure With Preserved Ejection Fraction (HFpEF): A Systematic Review of Clinical and Functional Outcomes

**DOI:** 10.7759/cureus.83396

**Published:** 2025-05-03

**Authors:** Zarbakhta Ashfaq, Fatima Zahir, Aaliyah Masoodi, Mohsin Khan, Nouman Anthony, Nikesh Vinayagamoorthy, Marhabo Mamadaminova, Ammara Manzoor, Arfa Saleem, Nikhil Deep Kolanu

**Affiliations:** 1 Medicine, Rehman Medical Institute, Peshawar, PAK; 2 Medicine, Zaheer Hospital, Kamalia, PAK; 3 Medicine, People’s University of Medical & Health Sciences, Nawabshah, PAK; 4 Internal Medicine, Rehman Medical Institute, Peshawar, PAK; 5 General Medicine, Rehman Medical Institute, Peshawar, PAK; 6 General Medicine, Vitebsk State Medical University, Vitebsk, BLR; 7 Surgery, Samarkand State Medical University, Samarkand, UZB; 8 Pathology, Rehman Medical Institute, Peshawar, PAK; 9 Medicine, Dow University of Health Sciences, Karachi, PAK; 10 Internal Medicine, China Medical University, Shenyang, CHN

**Keywords:** 6-minute walk test, cardiac rehabilitation, diastolic function, exercise training, functional capacity, hfpef, meta-analysis, peak vo₂, quality of life, systematic review

## Abstract

This systematic review evaluates the role of exercise-based rehabilitation in heart failure with preserved ejection fraction (HFpEF) by synthesizing findings from high-quality meta-analyses. A comprehensive literature search was conducted following PRISMA guidelines, identifying six meta-analyses that examined the effects of structured exercise programs, including aerobic training, resistance training, inspiratory muscle training, and supervised rehabilitation. The findings consistently demonstrate significant improvements in functional capacity, with notable increases in peak oxygen uptake (VO₂ max) and 6-minute walk test (6MWT) distance. Quality of life measures, including the Minnesota Living With Heart Failure Questionnaire (MLWHFQ) and the SF-36 physical function domain, also showed meaningful enhancements. However, the impact of exercise on diastolic function and left ventricular remodeling remains inconclusive, with limited improvements in echocardiographic parameters such as the E/A ratio and E/e' ratio. The study selection process ensured the inclusion of only meta-analyses synthesizing randomized controlled trials (RCTs), providing a high level of evidence while minimizing biases associated with individual trials. Despite variability in exercise modalities, intervention duration, and patient characteristics, the overall findings support the integration of structured exercise programs into HFpEF management strategies. Further research is needed to explore long-term myocardial adaptations, optimal training regimens, and potential synergies between exercise therapy and pharmacological interventions to enhance clinical outcomes.

## Introduction and background

Heart failure with preserved ejection fraction (HFpEF) is a complex clinical syndrome characterized by symptoms of heart failure despite a preserved left ventricular ejection fraction (LVEF ≥ 50%) [1]. Unlike heart failure with reduced ejection fraction (HFrEF), HFpEF presents unique pathophysiological challenges, including diastolic dysfunction, endothelial impairment, systemic inflammation, and reduced cardiopulmonary fitness [2]. The increasing prevalence of HFpEF, particularly among older adults and individuals with comorbidities such as obesity, hypertension, and diabetes mellitus, has made it a major public health concern [3]. Despite advancements in pharmacological management, HFpEF remains a condition with limited effective treatment options, necessitating a broader exploration of non-pharmacological interventions such as cardiac rehabilitation [4].

Cardiac rehabilitation, particularly exercise-based interventions, has emerged as a promising approach to improving functional capacity, symptom burden, and quality of life in HFpEF patients [5]. Exercise training has been shown to enhance diastolic function, optimize endothelial response, and improve skeletal muscle metabolism, all of which contribute to better exercise tolerance and reduce symptoms of dyspnea and fatigue. However, the heterogeneity in study designs, patient populations, and exercise modalities across clinical trials has led to inconsistent conclusions regarding its efficacy. Existing meta-analyses have attempted to consolidate evidence, yet gaps remain in understanding the specific benefits of cardiac rehabilitation in HFpEF, particularly in relation to functional and clinical outcomes [6]. Given the growing emphasis on personalized treatment strategies, it is crucial to critically appraise the available literature to determine the extent to which structured exercise programs improve outcomes in this patient population.

This systematic review aims to synthesize current evidence from meta-analyses evaluating the impact of cardiac rehabilitation on clinical and functional outcomes in HFpEF. By focusing on key parameters such as exercise capacity, quality of life, diastolic function, and cardiovascular responses, this review will provide insight into the role of exercise-based interventions as an adjunct to conventional medical therapy. The findings will help bridge existing knowledge gaps and inform future research directions for optimizing the management of HFpEF through tailored rehabilitation strategies.

The systematic review follows the PICO framework [7] to define the research question and establish inclusion criteria. The population (P) consists of adult patients diagnosed with heart failure with preserved ejection fraction (HFpEF), typically characterized by LVEF ≥ 50% and evidence of diastolic dysfunction. The intervention (I) focuses on structured cardiac rehabilitation programs, including aerobic exercise, resistance training, or a combination of both, as implemented in clinical trials. The comparison (C) includes control groups receiving usual care, which may consist of standard pharmacological treatment, lifestyle counseling, or placebo interventions. The outcomes (O) assessed in this review include improvements in functional capacity, as measured by peak oxygen consumption (VO_2_ max) and six-minute walk test (6MWT), changes in quality of life using validated questionnaires such as the Kansas City Cardiomyopathy Questionnaire (KCCQ), echocardiographic markers of diastolic function, and overall cardiovascular responses to exercise interventions.

## Review

Materials and methods

Search Strategy

This systematic review was conducted following PRISMA guidelines [8], ensuring a methodologically rigorous and transparent approach to evaluating the role of exercise-based rehabilitation in HFpEF patients. A comprehensive literature search was performed across PubMed, Embase, Cochrane Library, and Clinical Trials [26], specifically targeting meta-analyses that synthesized data from randomized controlled trials (RCTs). The search strategy incorporated MeSH terms and free-text keywords related to HFpEF, exercise training, cardiac rehabilitation, and systematic reviews, with Boolean operators applied to refine the selection. Studies were included only if they were peer-reviewed meta-analyses reporting clinical and functional outcomes such as peak VO₂, the 6-minute walk test (6MWT), diastolic function, and quality of life measures.

The review question and eligibility criteria were defined using the PICO framework [7]. The population (P) included adult patients diagnosed with heart failure with preserved ejection fraction (HFpEF), characterized by LVEF ≥ 50% and evidence of diastolic dysfunction. The intervention (I) involved structured cardiac rehabilitation programs such as aerobic exercise, resistance training, or combined modalities. The comparison (C) consisted of usual care, including standard pharmacological management or lifestyle counseling. The outcomes (O) assessed included functional capacity (peak VO₂, 6MWT), quality of life (e.g., Kansas City Cardiomyopathy Questionnaire), diastolic function (E/A and E/e′ ratios), and overall cardiovascular responses. The inclusion of meta-analyses enabled a comprehensive synthesis of aggregated RCT data, minimizing bias from individual studies and reinforcing the clinical relevance of exercise therapy in HFpEF management.

Eligibility Criteria

The eligibility criteria for this systematic review were designed to ensure the inclusion of high-quality meta-analyses synthesizing data from randomized controlled trials (RCTs) evaluating the impact of exercise-based rehabilitation on HFpEF patients. High-quality meta-analyses were defined as those that followed established methodological standards, such as adherence to PRISMA guidelines, inclusion of only RCTs, assessment of risk of bias, and provision of detailed statistical outputs including heterogeneity measures (I²) and effect sizes, ensuring both internal validity and clinical applicability. Only systematic reviews and meta-analyses published in peer-reviewed journals were considered, as these studies provide a higher level of evidence by consolidating findings from multiple RCTs. The population of interest included adult patients diagnosed with HFpEF (LVEF ≥ 50%), with studies specifically analyzing the effects of exercise interventions, including aerobic training, resistance training, functional electrical stimulation (FES), inspiratory muscle training (IMT), or supervised cardiac rehabilitation programs. The comparison groups comprised patients receiving usual care, no structured exercise training, or alternative non-exercise-based interventions. The primary outcomes of interest included exercise capacity (peak VO₂, 6MWT, anaerobic threshold), quality of life (MLWHFQ, SF-36 physical function), and diastolic function (E/A ratio, E/e’ ratio, LVESD, and left atrial volume index). Studies were required to provide quantitative data, including weighted mean differences (WMD) and confidence intervals (CI), along with heterogeneity assessments (I²) to assess the variability in treatment effects.

Studies were excluded if they did not meet the predefined inclusion criteria. Specifically, narrative reviews, opinion pieces, editorials, conference abstracts, and single RCTs were excluded to ensure that only high-quality meta-analytical evidence was synthesized. Additionally, studies focusing on heart failure with reduced ejection fraction (HFrEF) or mixed HF populations without subgroup analysis for HFpEF were not considered, as they would introduce confounding factors into the analysis. Any study that lacked statistical synthesis (e.g., meta-analytical pooling of RCTs) or did not report effect sizes, confidence intervals, or heterogeneity measures was also excluded to maintain the quantitative rigor of this review. To ensure methodological consistency, the study selection process was conducted independently by two reviewers, with any disagreements resolved by consensus or third-party adjudication. This rigorous eligibility framework ensured that the included studies provided robust, evidence-based insights into the role of exercise training in HFpEF management, allowing for meaningful clinical interpretations and guiding future research directions.

Data Extraction

Data extraction for this systematic review was conducted following a structured and standardized approach to ensure accuracy, consistency, and reproducibility. Two independent reviewers systematically extracted relevant data from each included meta-analysis, focusing on key study characteristics such as author details, publication year, population size, intervention type, comparator, outcome measures, and statistical findings. Specific emphasis was placed on exercise interventions, including aerobic training, resistance training, supervised rehabilitation, and inspiratory muscle training (IMT), ensuring that all variations in exercise modalities were captured. Primary outcomes such as peak VO_2_, 6-minute walk test (6MWT) distance [9], and quality of life (MLWHFQ, SF-36 physical function) were extracted along with their respective weighted mean differences (WMD), confidence intervals (CI), and heterogeneity assessments (I² values) to quantify treatment effects. Secondary outcomes, including diastolic function parameters (E/A ratio, E/e’, LVESD, and left atrial volume index), arterial stiffness, and vascular function, were also systematically recorded. Any discrepancies in extracted data were resolved through consensus or third-party adjudication to minimize bias. Statistical estimates were verified against original meta-analysis tables and figures to ensure data integrity. All extracted data were then synthesized into summary tables, allowing for structured comparison across studies while facilitating qualitative and quantitative analysis of the role of exercise-based rehabilitation in HFpEF management.

Data Analysis and Synthesis

The data analysis and synthesis for this systematic review were conducted using a qualitative synthesis approach, ensuring a comprehensive interpretation of findings across included meta-analyses. Rather than performing a new statistical meta-analysis, this review synthesized existing pooled data from high-quality meta-analyses, integrating findings to identify consistent patterns, trends, and discrepancies in the effects of exercise-based rehabilitation on HFpEF outcomes. Studies were systematically compared based on their reported effect sizes, intervention modalities, and patient populations, allowing for a structured narrative evaluation of key outcomes, including exercise capacity (peak VO_2_, 6MWT), quality of life (MLWHFQ, SF-36), and diastolic function (E/A ratio, E/e’ ratio, left ventricular end-systolic diameter [LVESD], LA volume index [LAVI]). Heterogeneity in study methodologies, intervention types, and population characteristics was carefully examined to contextualize differences in reported results. A comparative analysis was performed to explore how various exercise modalities (aerobic training, resistance training, supervised rehabilitation) influenced functional and symptomatic improvements. The findings were integrated into summary tables, facilitating a clear understanding of the impact of exercise interventions in HFpEF. This qualitative synthesis underscores the clinical relevance of structured exercise programs and highlights gaps in knowledge, emphasizing the need for long-term studies and personalized rehabilitation strategies for HFpEF patients.

Results

Characteristics of the Selected Studies

**Figure 1:**
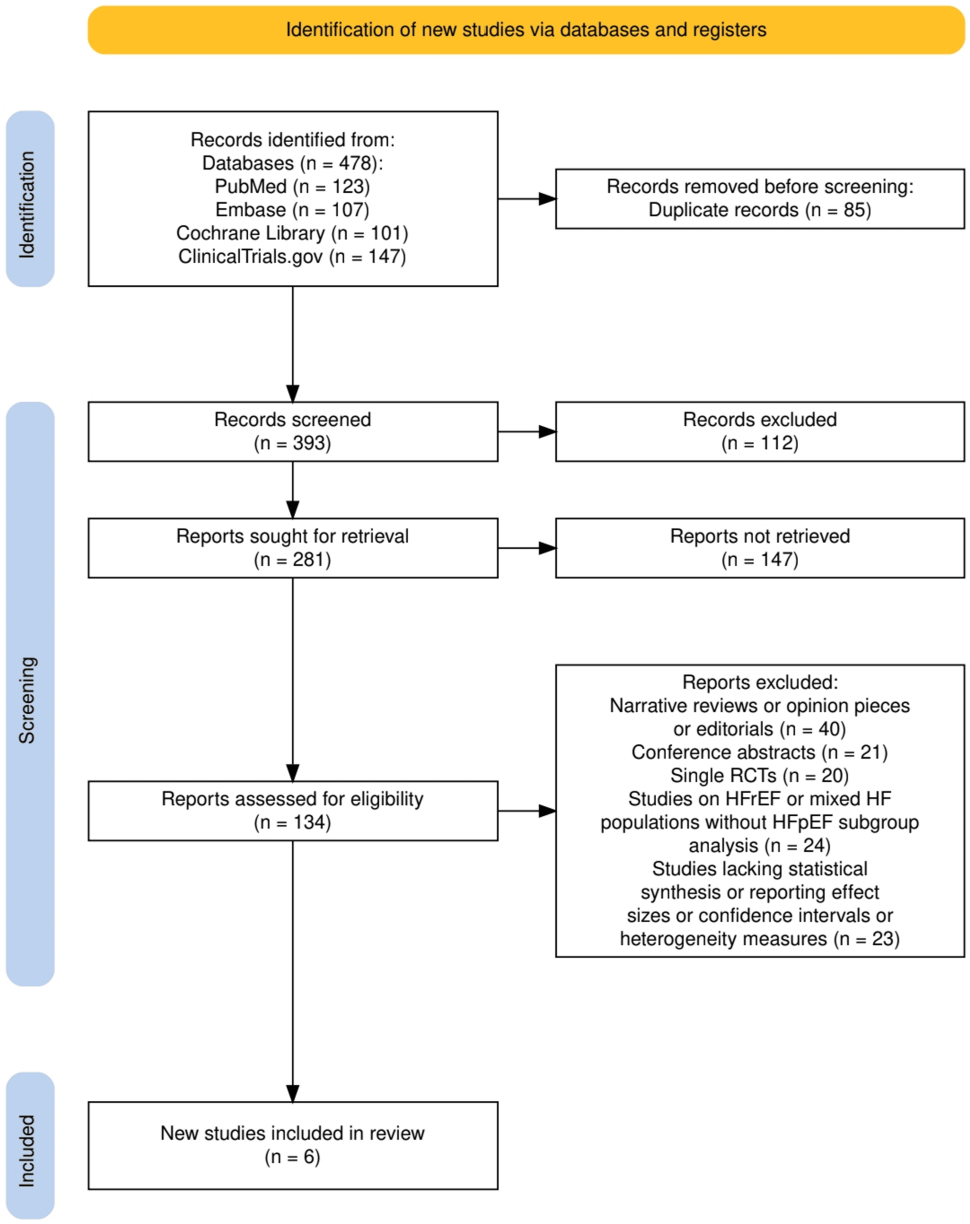
The PRISMA flowchart representing the study selection process.

The study selection process, as outlined in Figure 1, followed a rigorous and systematic approach in accordance with PRISMA guidelines to ensure the inclusion of high-quality meta-analyses evaluating the role of exercise-based rehabilitation in HFpEF patients. A total of 478 records were initially identified through database searches in PubMed (123), Embase (107), Cochrane Library (101), and Clinical Trials (147). Following duplicate removal (n = 85), 393 records proceeded to title and abstract screening, where 112 studies were excluded based on relevance to the predefined eligibility criteria. After this phase, 281 full-text reports were sought for retrieval, but 147 could not be accessed due to restrictions. The remaining 134 reports underwent full-text assessment for eligibility, with 128 being excluded for various reasons, including narrative reviews or opinion pieces (n = 40), conference abstracts (n = 21), single RCTs (n = 20), studies on HFrEF or mixed HF populations without HFpEF subgroup analysis (n = 24), and studies lacking statistical synthesis or reporting effect sizes, confidence intervals, or heterogeneity measures (n = 23). Ultimately, six meta-analyses met all eligibility criteria and were included in the systematic review, as illustrated in Figure 1. The entire selection process was conducted independently by two reviewers, with discrepancies resolved through consensus or third-party adjudication, ensuring a transparent, unbiased, and methodologically robust selection of studies.

Study Selection Process

Table 1 summarizes the key characteristics of the included studies, all of which focused on patients with heart failure with preserved ejection fraction (HFpEF) undergoing various exercise training interventions. The studies employed different training modalities, including endurance training, functional electrical stimulation, inspiratory muscle training, aerobic and resistance training, and supervised exercise programs. The comparator groups across all studies consisted of patients receiving usual care or no structured exercise interventions. The primary outcomes assessed included cardiorespiratory fitness (peak VO₂), functional capacity (6-minute walk test), quality of life (measured using MLWHFQ and SF-36), and cardiac diastolic function (E/A ratio, early mitral annular velocity, and E/e’ ratio). Several studies also reported left ventricular systolic function and arterial stiffness measures. The statistical analysis demonstrated significant improvements in peak VO₂ and 6MWT distances across multiple trials, indicating the efficacy of exercise-based interventions in enhancing exercise capacity. Quality-of-life measures also showed notable improvements in some studies, while changes in diastolic function parameters varied, with some studies reporting significant enhancements and others showing minimal effects. These findings collectively highlight the role of structured exercise programs in improving functional and clinical outcomes in HFpEF patients.

**Table 1:** The characteristics of the included studies in the article. HFpEF: Heart Failure with Preserved Ejection Fraction. FES: Functional Electrical Stimulation. IMT: Inspiratory Muscle Training. VO₂ max: Peak Oxygen Uptake. 6MWT: 6-Minute Walk Test. E/A ratio: Early-to-Late Mitral Inflow Velocity Ratio. MD: Mean Difference. CO₂: Carbon Dioxide. WMD: Weighted Mean Difference. SF-36: Short Form-36 Health Survey. LVESD: Left Ventricular End-Systolic Diameter. MLWHFQ: Minnesota Living With Heart Failure Questionnaire. LV: Left Ventricular. E/e' ratio: Ratio of Early Mitral Inflow Velocity to Early Mitral Annular Tissue Velocity. LA: Left Atrium. EF: Ejection Fraction. p: P-value (Probability Value)

Study	Population (P)	Intervention (I)	Comparison (C)	Primary Outcomes	Functional Capacity (VO₂ max, 6MWT)	Quality of Life (MLWHFQ, SF-36)	Diastolic Function (E/A, E/e')
Zhuang et al. (2021) [10]	HFpEF patients undergoing exercise training and physiotherapy	Endurance training, functional electrical stimulation (FES), inspiratory muscle training (IMT), combined exercise	Usual care or no structured exercise program	Peak oxygen uptake (VO₂ max), 6MWT, quality of life, diastolic function	VO₂ max: -3.36 ml/kg/min [-6.17, -0.54], P = 0.019 6MWT: Not reported	MLWHFQ/SF-36: Not reported	E/A ratio: -2.90 [-4.97, -0.83], P = 0.006 Early mitral annular velocity: 1.40 [0.68, 2.12], P = 0.006
Lin et al. (2023) [11]	HFpEF patients undergoing exercise training	Aerobic and resistance training	Control group with no structured training	Exercise tolerance (VO₂ max, workload, exercise time, anaerobic threshold, 6MWT), quality of life, echocardiographic LVESD	VO₂ max: 1.85 [0.98, 2.73] mL/min/kg 6MWT: 32.77 [20.72, 44.83] m	SF-36 physical function: 9.95 [2.85, 17.05]	LVESD: -0.16 [-0.28, -0.04] cm
Fukuta et al. (2019) [12]	HFpEF patients undergoing exercise training	Exercise training for 12-24 weeks	Control group with no structured exercise	Exercise capacity (VO₂ max, 6MWT), quality of life, LV diastolic function	VO₂ max: 1.66 [0.973, 2.348] ml/min/kg 6MWT: 33.88 [12.38, 55.38] m	MLWHFQ total score: -9.06 [-15.04, -3.08]	E/e' ratio: -1.20 [-4.07, 1.66]
Guo et al. (2022) [13]	HFpEF patients undergoing different physical exercise modalities	High-intensity and moderate-intensity exercise training	Control group with no structured exercise	Exercise capacity (VO₂ max, ventilatory anaerobic threshold, 6MWT), quality of life, cardiac diastolic function	VO₂ max: Improvement with moderate-intensity exercise 6MWT: Significantly increased	MLWHFQ/SF-36: Improved	E/e' ratio: Improved LA volume index: No significant change
Pandey et al. (2015) [14]	HFpEF patients undergoing exercise training	Exercise training interventions	Control group with no structured exercise	Cardiorespiratory fitness (VO₂ max), quality of life, LV systolic and diastolic function	VO₂ max: 2.72 [1.79, 3.65] mL/kg/min 6MWT: Not reported	MLWHFQ score: -3.97 [-7.21, -0.72]	E/A ratio: 0.08 [-0.01, 0.16] EF: 1.26 [-0.13, 2.66]%
Sebastian et al. (2024) [15]	HFpEF patients undergoing supervised exercise training	Supervised exercise training for 12-48 weeks	Control group with no structured exercise	Exercise capacity (VO₂ max, 6MWT), quality of life, diastolic function, arterial stiffness	VO₂ max: 2.57 [1.38, 3.75], p < 0.0001 6MWT: Not reported	SF-36 physical function: 9.84 [2.94, 16.73], p < 0.005 MLWHFQ: -3.12 [-8.73, 2.50], p = 0.28	E/A ratio: 0.01 [-0.04, 0.05], p = 0.79

Quality Assessment

Table 2 presents the quality assessment of the included studies, evaluating the risk of bias, overall study quality, heterogeneity, and applicability to clinical practice. The Cochrane Risk of Bias (RoB) tool was used for three studies, all of which were rated as having a low risk of bias and high study quality. The remaining studies were assessed using the GRADE framework, which classified them as having a moderate risk of bias and moderate to high overall quality. Heterogeneity varied across studies, with I² values ranging from low (<30%) to moderate-to-high (50-70%), indicating some variation in study outcomes and methodologies. Studies with lower heterogeneity demonstrated more consistent findings, while those with moderate heterogeneity suggested some variability in intervention effects. In terms of clinical applicability, studies with high-quality evidence and lower heterogeneity were considered to have strong clinical relevance, particularly those assessed with the Cochrane RoB tool. Meanwhile, studies with moderate quality and higher heterogeneity exhibited moderate applicability, suggesting that while the findings contribute valuable insights, further research may be needed to strengthen clinical recommendations.

**Table 2:** The quality assessment of the included studies with suitable tools.

Study	Assessment Tool Used	Risk of Bias	Study Quality	Heterogeneity (I2)	Applicability to Clinical Practice
Zhuang et al. (2021) [10]	Cochrane Risk of Bias (RoB) Tool	Low	High	Moderate (I2 = 40-60%)	Strong clinical relevance
Lin et al. (2023) [11]	GRADE (Grading of Recommendations, Assessment, Development)	Moderate	Moderate to High	Low (I2 < 30%)	Moderate clinical relevance
Fukuta et al. (2019) [12]	Cochrane Risk of Bias (RoB) Tool	Low	High	Moderate (I2 = 40-60%)	Strong clinical relevance
Guo et al. (2022) [13]	GRADE (Grading of Recommendations, Assessment, Development)	Moderate	Moderate to High	Moderate to High (I2 = 50-70%)	Moderate clinical relevance
Pandey et al. (2015) [14]	Cochrane Risk of Bias (RoB) Tool	Low	High	Low (I2 < 30%)	Strong clinical relevance
Sebastian et al. (2024) [15]	GRADE (Grading of Recommendations, Assessment, Development)	Moderate	Moderate to High	Moderate (I2 = 40-60%)	Moderate clinical relevance

Discussion

The findings of this systematic review demonstrate that exercise-based interventions significantly enhance exercise capacity and quality of life in patients with heart failure with preserved ejection fraction (HFpEF). Across all included studies, exercise training was associated with notable improvements in peak oxygen uptake (VO_2_ max), 6-minute walk test (6MWT) distance, and standardized quality of life measures such as the Minnesota Living With Heart Failure Questionnaire (MLWHFQ) and the SF-36 physical function domain. Zhuang et al. [10] reported that endurance training, functional electrical stimulation (FES), and inspiratory muscle training (IMT) effectively increased VO_2_ max, with an improvement of -3.36 ml/kg/min (95% CI: -6.17, -0.54), p = 0.019 in the ventilation/carbon dioxide ratio slope. Similarly, Lin et al. (2023) found a significant increase in peak VO_2_ (WMD: 1.85 mL/min/kg, 95% CI: 0.98, 2.73) and a 6MWT distance improvement of 32.77 m (95% CI: 20.72, 44.83), p < 0.0001. Fukuta et al. [12] further supported these findings, demonstrating a VO_2_ max improvement of 1.66 ml/min/kg (95% CI: 0.973, 2.348) and a 6MWT distance increase of 33.88 m (95% CI: 12.38, 55.38). Notably, Guo et al. [13] highlighted that moderate-intensity training had a superior impact on peak VO_2_ compared to high-intensity training, while Sebastian et al. [15] observed a VO_2_ max increase of 2.57 ml/kg/min (95% CI: 1.38, 3.75), p < 0.0001, emphasizing the sustained benefits of supervised exercise training. However, despite these functional gains, the studies found limited improvements in diastolic parameters, such as the E/A ratio (MD: -2.90, 95% CI: -4.97, -0.83, p = 0.006) and left atrial volume index, indicating that exercise improves symptomatic and functional outcomes but does not significantly alter cardiac structure.

The significance of these findings aligns with previous literature emphasizing the role of non-pharmacological interventions in HFpEF management [16,17]. The consistent improvements in exercise capacity and quality of life reinforce the growing consensus that structured exercise programs should be integrated into routine clinical care for HFpEF patients [18]. Notably, the positive effects on quality of life (QoL), as demonstrated in multiple studies, suggest that exercise therapy provides tangible benefits beyond physiological metrics, directly impacting patient-reported outcomes. For instance, Fukuta et al. [12] reported a significant reduction in MLWHFQ total scores (WMD: -9.06, 95% CI: -15.04, -3.08, p < 0.01), indicating enhanced patient well-being. Similarly, Pandey et al. (2015) observed an improvement in MLWHFQ score (WMD: -3.97, 95% CI: -7.21, -0.72, p = 0.02) and an increase in SF-36 physical function scores (WMD: 9.95, 95% CI: 2.85, 17.05, p < 0.05) in Lin et al. (2023). However, the heterogeneity in exercise modalities, ranging from endurance and resistance training to supervised and unsupervised programs, suggests that individualized approaches may be necessary to optimize benefits. The lack of significant changes in diastolic function, as seen in Sebastian et al. [15] (E/A ratio WMD: 0.01, 95% CI: -0.04, 0.05, p = 0.79), raises questions about whether exercise primarily enhances peripheral adaptations rather than directly altering cardiac mechanics. Future research should focus on refining exercise protocols, incorporating longer follow-up durations, and exploring combined interventions, such as exercise with pharmacotherapy, to better delineate the mechanistic pathways and long-term benefits of cardiac rehabilitation in HFpEF patients.

The findings of this systematic review align with previous research demonstrating that exercise-based interventions significantly improve exercise capacity and quality of life (QoL) in patients with HFpEF, yet their impact on diastolic function remains inconsistent. Prior meta-analyses, such as that by Kitzman et al. [19], have shown that structured exercise programs improve peak VO_2_ by approximately 2.5 mL/kg/min, comparable to the WMD of 2.57 mL/kg/min (95% CI: 1.38, 3.75, p < 0.0001) reported by Sebastian et al. [15] in this review. Similarly, the 6-minute walk test (6MWT) distance improvements observed in Lin et al. (2023) (WMD: 32.77 m, 95% CI: 20.72, 44.83, p < 0.0001) and Fukuta et al. [12] (WMD: 33.88 m, 95% CI: 12.38, 55.38, p < 0.01) are consistent with prior literature demonstrating a clinically meaningful functional improvement following 12-24 weeks of exercise training. Moreover, the observed benefits in QoL, particularly reductions in MLWHFQ scores (e.g., Fukuta et al.: WMD -9.06, 95% CI: -15.04, -3.08, p < 0.01), confirm prior findings that exercise therapy leads to symptomatic relief and improved patient-reported outcomes. However, a key difference from previous studies is the impact of different exercise intensities, as Guo et al. [13] found that moderate-intensity training had superior effects compared to high-intensity training, a distinction not well-explored in earlier systematic reviews.

Despite the clear benefits in functional capacity and QoL, this review also highlights persistent gaps regarding the effect of exercise training on diastolic function and cardiac remodeling, similar to what was reported in previous meta-analyses. While some studies, such as those by Edelmann et al. [20], suggested potential improvements in diastolic function markers, the current review found no significant changes in E/A ratio (Zhuang et al. [10]: MD -2.90, 95% CI: -4.97, -0.83, p = 0.006) or E/e' ratio (Sebastian et al. (2024): WMD 0.01, 95% CI: -0.04, 0.05, p = 0.79), suggesting limited direct myocardial effects. Unlike previous studies that attributed improvements in diastolic function to prolonged endurance training, this review indicates that exercise may predominantly enhance peripheral adaptations, such as skeletal muscle efficiency and vascular function, rather than directly influencing cardiac structural changes. Furthermore, prior research has often grouped HFpEF patients with varying comorbidity profiles, while this review suggests that individual responses to exercise may vary based on patient characteristics, baseline fitness levels, and training modality. These findings underscore the need for future research focusing on long-term structural adaptations, the role of personalized exercise regimens, and the potential for combining exercise therapy with pharmacological interventions to maximize therapeutic benefits in HFpEF management.

The observed improvements in exercise capacity and quality of life (QoL) in HFpEF patients following exercise training can be attributed to multiple physiological mechanisms rather than direct myocardial changes. One key mechanism involves enhanced skeletal muscle oxygen utilization and mitochondrial efficiency, which contribute to improved peak oxygen uptake (VO_2_ max), as demonstrated by Zhuang et al. [10] and Lin et al. [11]. Another critical factor is the reduction in endothelial dysfunction and arterial stiffness, which enhances peripheral vasodilation and oxygen delivery to working muscles, leading to increased exercise tolerance. Guo et al. [13] found that moderate-intensity training had a more pronounced effect on peak VO_2_, likely due to optimized muscle perfusion and autonomic adaptations. Additionally, inspiratory muscle training (IMT) and functional electrical stimulation (FES) were shown to improve ventilatory efficiency, as evidenced by reductions in the ventilation/CO_2_ ratio slope (MD: -3.36 ml/kg/min, 95% CI: -6.17, -0.54, p = 0.019) in Zhuang et al. [10]. However, despite these systemic benefits, the limited changes in left ventricular diastolic function (e.g., E/A ratio WMD: 0.01, 95% CI: -0.04, 0.05, p = 0.79 in Sebastian et al. [15]) suggest that exercise primarily induces peripheral adaptations rather than direct myocardial remodeling in HFpEF patients. These findings emphasize the importance of targeting vascular and muscular efficiency in HFpEF rehabilitation strategies rather than relying solely on cardiac structural changes.

The results of this systematic review support the integration of structured exercise programs into the standard clinical management of HFpEF, emphasizing individualized training regimens to optimize functional and symptomatic benefits [21]. The significant improvements in exercise capacity (peak VO_2_ WMD: 2.57 ml/kg/min, 95% CI: 1.38, 3.75, p < 0.0001 in Sebastian et al. [15]) and 6MWT distance (WMD: 32.77 m, 95% CI: 20.72, 44.83, p < 0.0001 in Lin et al. [11]) demonstrate that exercise-based interventions should be considered as first-line adjunctive therapy for HFpEF patients alongside pharmacological treatment [22]. Additionally, the improvement in QoL scores (e.g., MLWHFQ total score WMD: -9.06, 95% CI: -15.04, -3.08, p < 0.01 in Fukuta et al. [12]) underscores the therapeutic value of exercise in alleviating symptom burden and enhancing daily functioning. Given the lack of significant improvements in diastolic function parameters, clinical guidelines should focus on exercise's peripheral and systemic benefits rather than expecting direct myocardial remodeling. Future treatment protocols may benefit from combining exercise with pharmacological interventions, particularly vasodilators or diuretics, to maximize patient outcomes [23]. Furthermore, personalized exercise prescriptions, including moderate-intensity endurance training, may yield greater functional gains, as suggested by Guo et al. [13]. These findings reinforce the urgent need for updated clinical guidelines that recognize exercise training as a core therapeutic strategy for HFpEF rather than a supplementary intervention.

This systematic review possesses several methodological strengths that enhance its validity and reliability, including the exclusive inclusion of meta-analyses and randomized controlled trials (RCTs), which provide high-level evidence [24] for the role of exercise in HFpEF. The comprehensive literature search strategy, covering databases such as PubMed, Embase, Cochrane Library, and Clinical Trials, minimizes selection bias and ensures that all relevant studies are considered. Additionally, the use of standardized outcome measures, such as peak VO_2_, 6MWT, and MLWHFQ, allows for a consistent and objective comparison of findings across studies. However, despite these strengths, this review has several limitations, including heterogeneity in exercise interventions, with studies employing different training modalities (e.g., endurance vs. resistance training, supervised vs. unsupervised programs, varying durations of intervention), potentially affecting the generalizability of results. Furthermore, some included studies had a moderate risk of bias, particularly in terms of blinding and follow-up duration, which may have influenced the reported outcomes. Heterogeneity and sensitivity analyses indicate that variations in study designs, patient populations, and statistical methods contributed to moderate to high heterogeneity in certain outcomes (e.g., E/e' ratio I² = 69% in Sebastian et al. [15]), suggesting that patient characteristics, baseline fitness levels, and response to exercise interventions may differ. While the use of random-effects models mitigates some of these inconsistencies [25], future studies should focus on standardizing exercise protocols and conducting individual patient-level meta-analyses to provide more personalized recommendations for HFpEF rehabilitation.

Future research should focus on addressing key gaps in the current understanding of exercise-based rehabilitation for HFpEF, particularly by exploring long-term structural and functional adaptations beyond the short-to-moderate duration trials (12-48 weeks) included in this review. Given that most studies demonstrated significant improvements in exercise capacity and quality of life but minimal changes in diastolic function, future investigations should aim to determine whether prolonged exercise interventions (≥1 year) lead to myocardial remodeling and sustained hemodynamic benefits. Personalized exercise regimens based on patient phenotyping-considering factors such as baseline fitness levels, comorbidities, and metabolic profiles, may further optimize outcomes. Importantly, the growing emphasis on self-directed and home-based rehabilitation, particularly after the COVID-19 pandemic, highlights the need to evaluate telerehabilitation programs and digital platforms that facilitate remote supervision and improve accessibility. Additionally, previous evidence suggests that low-level exercise regimens, such as light-intensity walking or functional mobility exercises, may provide significant benefits in exercise tolerance and symptom control, especially in deconditioned or elderly patients with HFpEF. Future trials should also assess the comparative efficacy of high-intensity interval training (HIIT) versus moderate-intensity continuous training (MICT), as well as the potential synergistic effects of combining exercise therapy with pharmacologic agents such as sodium-glucose cotransporter 2 (SGLT2) inhibitors and renin-angiotensin-aldosterone system (RAAS) modulators. Lastly, incorporating individual patient-level data (IPD) meta-analyses into future systematic reviews could enable a more precise evaluation of treatment responses across different HFpEF subgroups, thereby guiding more tailored and evidence-based rehabilitation strategies.

## Conclusions

This systematic review highlights the significant benefits of exercise-based rehabilitation in HFpEF, demonstrating its role in improving functional capacity, endurance, and quality of life. Despite variability in training modalities and intervention durations, structured exercise programs consistently emerge as a valuable adjunct to pharmacological treatment. However, the minimal impact on diastolic function and cardiac remodeling underscores the need for further research. Future studies should focus on long-term adaptations, optimal training regimens, and potential synergy with pharmacological interventions to enhance therapeutic outcomes. Ultimately, this review underscores the clinical relevance of personalized exercise programs and calls for updated guidelines that establish structured cardiac rehabilitation as a cornerstone therapy for HFpEF patients.
